# P2X Receptor-Dependent Erythrocyte Damage by α-Hemolysin from *Escherichia* *coli* Triggers Phagocytosis by THP-1 Cells

**DOI:** 10.3390/toxins5030472

**Published:** 2013-03-05

**Authors:** Steen K. Fagerberg, Marianne Skals, Jens Leipziger, Helle A. Praetorius

**Affiliations:** Department of Biomedicine, Physiology, Aarhus University, Ole Worms Allé 4, 8000 Aarhus C, Building 1160, Aarhus 8000, Denmark; E-Mails: steenfagerberg@gmail.com (S.K.F.); mgs@fi.au.dk (M.S.); leip@fi.au.dk (J.L.)

**Keywords:** phagocytosis, phosphatidyl serine, hemolysin *E.coli*, monocytes, hemolysis, P2X

## Abstract

The pore-forming exotoxin α-hemolysin from *E.* *coli* causes a significant volume reduction of human erythrocytes that precedes the ultimate swelling and lysis. This shrinkage results from activation of Ca^2+^-sensitive K^+^ (K_Ca_3.1) and Cl^−^ channels (TMEM16A) and reduced functions of either of these channels potentiate the HlyA-induced hemolysis. This means that Ca^2+^-dependent activation of K_Ca_3.1 and TMEM16A protects the cells against early hemolysis. Simultaneous to the HlyA-induced shrinkage, the erythrocytes show increased exposure of phosphatidylserine (PS) in the outer plasma membrane leaflet, which is known to be a keen trigger for phagocytosis. We hypothesize that exposure to HlyA elicits removal of the damaged erythrocytes by phagocytic cells. Cultured THP-1 cells as a model for erythrocytal phagocytosis was verified by a variety of methods, including live cell imaging. We consistently found the HlyA to very potently trigger phagocytosis of erythrocytes by THP-1 cells. The HlyA-induced phagocytosis was prevented by inhibition of K_Ca_3.1, which is known to reduce PS-exposure in human erythrocytes subjected to both ionomycin and HlyA. Moreover, we show that P2X receptor inhibition, which prevents the cell damages caused by HlyA, also reduced that HlyA-induced PS-exposure and phagocytosis. Based on these results, we propose that erythrocytes, damaged by HlyA-insertion, are effectively cleared from the blood stream. This mechanism will potentially reduce the risk of intravascular hemolysis.

## 1. Introduction

The limited lifespan of mammalian erythrocytes indirectly results from the lack of nuclei in this cell type. Thus, erythrocytes have an innate inability to renew cellular proteins, and maintain a normal cell homeostasis on a long-term basis. Another consequence of the absence of nuclei is that they cannot undergo regular apoptosis and, consequently, the normal pathway of clearing old or damaged cells does not immediately apply for mammalian erythrocytes. Nevertheless, aging erythrocytes are efficiently recognized by phagocytes and removed from the bloodstream. Loss of their biconcave shape—as seen in spherocytosis and sickle cell disease—is a major signal for erythrocyte clearance, primarily through monocytes/macrophages in the spleen (for a review, see [1]). We have recently shown that bacterial hemolysins have an enormous impact on the erythrocyte shape [2,3,4,5]. The pore-forming toxin α-hemolysin (HlyA) from *E.* *coli* does not only cause swelling and lysis of the erythrocytes. The toxin inflicts distinct biphasic volume changes, where the volume increase that leads to lysis is preceded by marked erythrocyte shrinkage. This shrinkage is triggered by the influx of Ca^2+^, which happens very quickly after insertion of HlyA into the membrane. This Ca^2+^ influx activates Ca^2+^-sensitive K^+^ channels (K_Ca_3.1) and Cl^−^ channels (TMEM16A) [3], which are thus responsible for the KCl efflux that results in the HlyA-induced volume reduction. Diminishing the function of either of these channels potentiate the toxin-induced hemolysis substantially, which implies that shrinkage protects the erythrocytes from early lysis. Interestingly, the HlyA-induced erythrocyte shrinkage is associated with phosphatidylserine (PS) exposure in the outer leaflet of the plasma membrane that is prevented when the erythrocyte shrinkage is blocked by K_Ca_3.1 inhibitors [3]. PS exposure and reduced cell volume have both been suggested to be a signal for erythrocyte clearance [6,7,8]. We therefore hypothesize that the damage insertion of HlyA inflicts on the erythrocyte makes them more disposed to be phagocytosed.

Here, we show that insertion of HlyA into the erythrocyte membrane is a potent signal for erythrocyte phagocytosis by THP-1 cells. Inhibiting the K_Ca_3.1 by TRAM34, which is known to decrease PS exposure, significantly reduced the phagocytosis of HlyA-exposed erythrocytes by THP-1 cells. P2 receptor blockers, which protect the erythrocytes against HlyA-induced cell damage, also caused a marked reduction of the HlyA-induced PS exposure. In parallel, short pre-incubation with the irreversible P2X receptor blocker oxATP partially prevented the HlyA-induced phagocytosis of the erythrocytes. The perspective of this finding is that the shrinkage seen in erythrocyte damaged by pore-forming toxins triggers phagocytosis. This will, in turn, prompt the elimination of the damaged erythrocytes from the blood stream, and potentially protect against intravascular hemolysis.

## 2. Results

**Figure 1:**
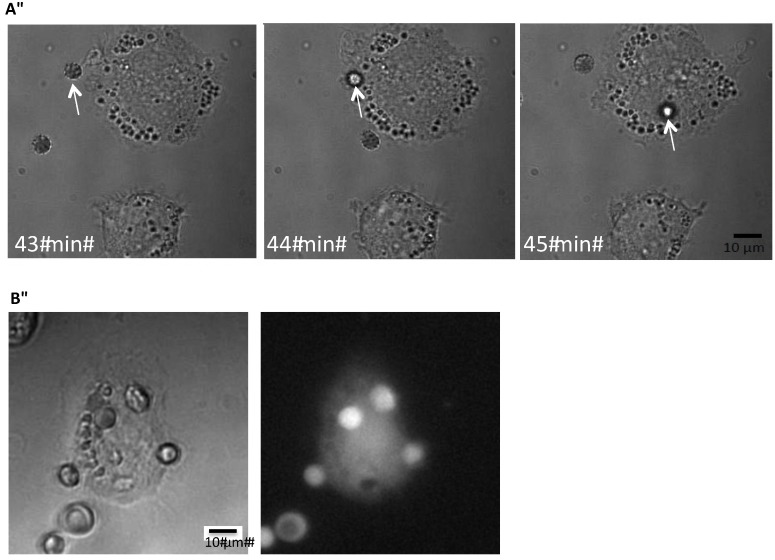
Phagocytosis of erythrocytes by THP-1 cells. (**A**) shows the time-course of phagocytosis of a single erythrocyte by a THP-1 cell grown on a coverslip but DIC; (**B**) shows corresponding pictures obtained by DIC or low light fluorescence of calcein-loaded human erythrocytes phagocytosed by a THP-1 cell grown on a coverslip.

The human macrophage/monocytic cell line THP-1 was used to investigate whether pore-forming toxins like HlyA renders erythrocytes more liable for recognition and phagocytosis. Initially, we wanted to establish a method to detect phagocytosis of erythrocytes by THP-1 cells. It is known that erythrocytes exposed to a Ca^2+^-ionophore makes them prone to be phagocytosed by monocytes and macrophages [9,10]. To visualize the process, we mounted THP-1 cells grown on a coverslip in an incubation chamber on an inverted microscope. Figure 1 shows the time-lapse of the phagocytosis of an erythrocyte exposed to ionomycin (1 μM) by a THP-1 cell. To be able to test whether HlyA is able to induce erythrocyte phagocytosis, we needed a reliable, easily quantifiable method to determine the phagocytosis. Our first question was whether the erythrocytes are actually phagocytosed or if they just stay attached to the plasma membrane of THP-1 cells. Therefore, we added erythrocytes subjected to ionomycin (1 μM) for 10 min to the THP-1 cells grown on a coverslip. After 60 min of incubation, the preparation was fixed, marked with an anti-hemoglobin antibody and inspected with structural illumination microscopy. By an overview of the preparation, erythrocytes were only found in connection with THP-1 cells. By inspecting various focus planes, the FITC-fluorescence from the erythrocytes was only localized inside the THP-1 cells. To illustrate this, we generated z-stacks from a plane where the erythrocyte was in focus and this plane was used as the reference plane for both the fluorescence and z-stacks of transmitted light images in differential interference contrast (DIC). For the fluorescence z-stacks, 30 planes of 0.2 μm were imaged on both sides of the erythrocyte and 50 planes were imaged of 0.2 μm for the DIC-stack. Afterwards the z-stacks were aligned after the reference plane. A representative example is shown in supplementary Figure 1A,B, where the side-view clearly shows that the anti-hemoglobin antibody staining is seen inside the cytoplasm of the THP-1 cells.

As the next step, we loaded the erythrocytes with the fluorescent probe fluo 4. The erythrocytes were again subjected to ionomycin (1 μM) for 10 min, before they were incubated with the THP-1 cells for 60 min. Consecutive images with DIC or fluorescence (488 nm) microscopy were taken of at least 100 THP-1 cells per preparation. The THP-1 cells were identified in the DIC images, and the ones that show fluorescence intensity over background was identified as positive. Ionomycin (1 μM), which is known to induce phagocytosis, increased the number of fluorescent positive THP-1 cells (Figure S1C,D, *p* = 0.05).

**Figure 2:**
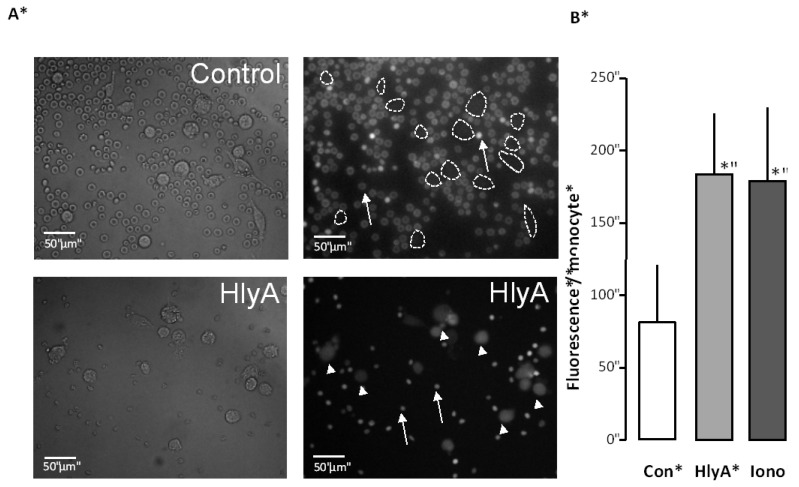
The effect of HlyA on erythrocyte phagocytosis by THP-1 cells. Differential count of THP-1 cells that have taken up the fluorescent probe calcein from calcein-AM loaded erythrocytes, either exposed to HlyA (10 min) at a concentration that cause 50% hemolysis after 60 min or ionomycin (1 μM) as a positive control. (**A**) presents images of the phagocytosis of the control erythrocytes (arrows show erythrocytes, lines show boundaries of two THP-1 cells) and erythrocytes exposed to HlyA (arrow show erythrocytes, arrowheads THP-1 cells); (**B**) shows the summarized data as mean ± SEM, *n* = 8). Asterisk denominates statistically the significance of the control with a *p*-value below 0.05.

To determine whether the fluorescence observed in the THP-1 cells actually originates from the erythrocytes, we used the Ca^2+^-sensing property of the fluorophore (fluo 4). We were able to observe that the fluorophore after phagocytosis, unlike the anti-hemoglobin antibody, was not confined to the engulfed erythrocytes but distributed diffusely in the cytosol of the THP-1 cells. This finding means that we could not immediately conclude that the increase in fluorescence of the THP-1 cells was caused by phagocytosis of fluorescent erythrocytes. If the increase in THP-1 cell fluorescence observed after ionomycin treatment comes from engulfed erythrocytes, the properties of fluo 4 should be conserved. To test this, we performed two types of experiments. First, we directly loaded THP-1 cells with fluo 4-AM (Figure S1E). We were able to determine that the THP-1 cells responded to extracellularly applied ATP (100 μM) by a substantial increase in [Ca^2+^]_i_. Afterwards, fluo 4-loaded erythrocytes, incubated with ionomycin (1 μM, 10 min) were added to a preparation of THP-1 cells grown on coverslips, incubated for 60 min at 37 °C and thereafter used for live cell microscopy. Adding ATP (100 μM) again produced a rapid transient increase in the intracellular fluorescence of the THP-1 cells. These data support that the increase in fluorescence observed in erythrocyte-exposed THP-1 cells actually is caused by fluo 4 introduced via erythrocyte phagocytosis.

This way reassured that our assay reports about the phagocytosis of erythrocytes, we tested whether HlyA would make the erythrocytes more prone to phagocytosis. In these experiments, the erythrocytes were loaded with calcein-AM to prevent the fluorescence intensity of the THP-1 cells to depend on changes in [Ca^2+^]_i_. Erythrocytes were incubated for 10 min at 37 °C with a HlyA concentration, which results in 50% hemolysis after 60 min of incubation. Figure 2 shows that this treatment was equally effective in potentiating erythrocyte phagocytosis by the THP-1 cells as ionomycin (1 μM). Figure 2A shows representative images. For the summarized data, the THP-1 cells were identified by DIC images and the regions of interest were placed over the cells. The bar graph shows the mean fluorescence detected over all cells.

**Figure 3:**
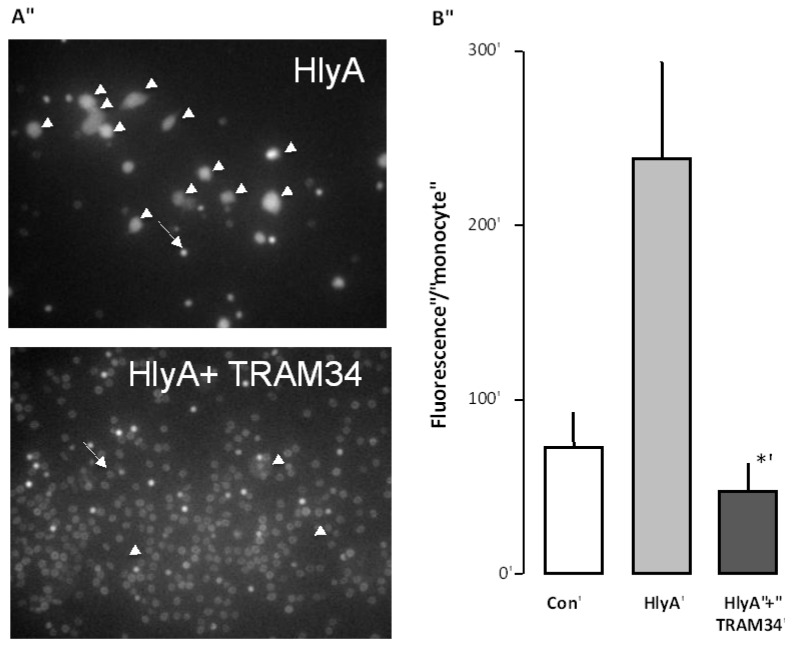
The effect of the K_Ca_3.1 inhibitor (TRAM34) on the HlyA-induced phagocytosis of human erythrocytes by THP-1 cells**.** (**A**) shows representative images of the HlyA-induced phagocytosis of human erythrocytes by THP-1 cells in the absence or presence of TRAM34 (10 μM). The arrow shows an erythrocyte the arrowheads show THP-1 cells; (**B**) shows the summarized data as mean ± SEM, *n* = 8. The asterisk shows a statistical significance from HlyA with a *p*-value below 0.05.

The phagocytosis of damaged erythrocytes has been suggested to be dependent partially on phosphatidylserine (PS) exposure in the outer leaflet of the erythrocytes [11]. We have previously shown that inhibition of the initial shrinkage of the erythrocytes induced by HlyA is prevented by inhibition of the Ca^2+^-sensitive K^+^ channel K_Ca_3.1 [3]. We could also show that this shrinkage was associated with an increased PS exposure, which could be prevented by inhibition of K_Ca_3.1 channels. Here, we tested whether inhibition of K_Ca_3.1 channels also would prevent the phagocytosis of erythrocytes. To this end, the erythrocytes were again incubated with calcein-AM for 60 min, and subjected to HlyA for 10 min, at a concentration that causes 50% hemolysis after 60 min, either in the absence or the presence of TRAM34 (10 μM). Afterwards, both HlyA and TRAM34 were washed away from the erythrocytes and they were added onto the THP-1 cells. The results clearly show that the presence of TRAM34 during the incubation with HlyA considerably reduced the phagocytosis otherwise induced by HlyA (Figure 3). These data are in agreement with PS as an important signal for phagocytosis.

To illustrate the significance of PS in the THP-1 cells object recognition, we used PS and PC-coated beads. These types of beads are commercially available in a non-fluorescent form and thus, we loaded the cells with calcein-AM for an easier detect of the engulfed beads. Figure S2A shows two representative images of THP-1 cells subjected to either PS or PC covered beads. The data summarized (Figure S2B) clearly shows that the PS-covered beads are taken up to a larger extent by the THP-1 cells.

**Figure 4:**
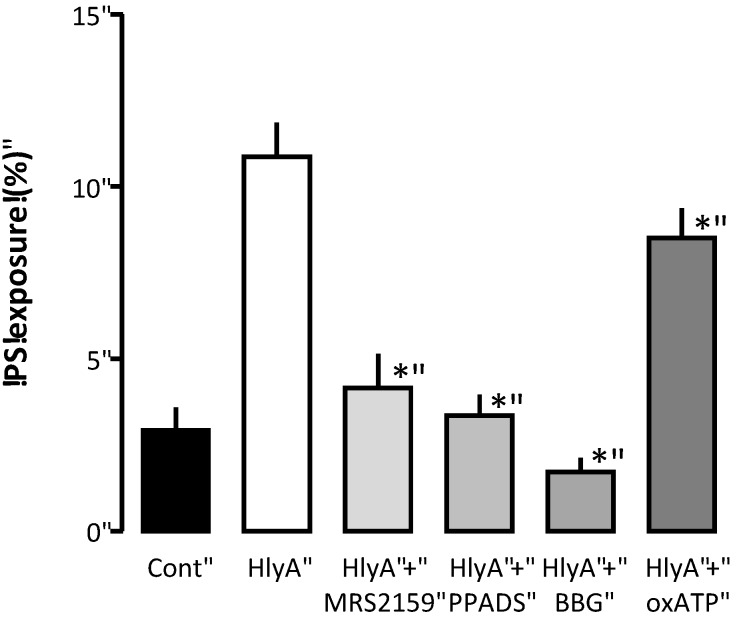
The effect of P2 receptor antagonists on HlyA-induced phosphatidylserine exposure of human erythrocytes. Human erythrocytes were exposed to HlyA at a very low concentration that did not cause any detectable lysis after 10 min. The HlyA-induced PS exposure measured as FITC-conjugated annexin V binding was prevented by inhibition of P2 receptor antagonists: MRS2159 (250 μM), PPADS (500 μM), BBG (3 μM) and oxATP (500 μM). The graph shows mean ± SEM, *n* = 5–7, asterisks illustrate a statistically significant difference from the control with a *p*-value below 0.05.

As the HlyA-induced hemolysis is greatly attenuated by P2 receptor inhibition, we tested whether blocking P2 receptors would also prevent PS exposure in the erythrocytes. In Figure 4, the PS exposure is measured by flow cytometry as an increase in the fluorescence of FITC-conjugated annexin V binding. To induce PS exposure, the erythrocytes were subjected to a concentration of HlyA (1 ng mL^−1^), which did not produce any detectable hemolysis within 30 min. At this HlyA concentration, ≈10% of the erythrocytes showed PS exposure compared to ≈3% in the control situation. The P2 receptor antagonists PPADS, BBG, MRS2159 and oxATP significantly reduced the PS exposure (*p* < 0.05). 

**Figure 5:**
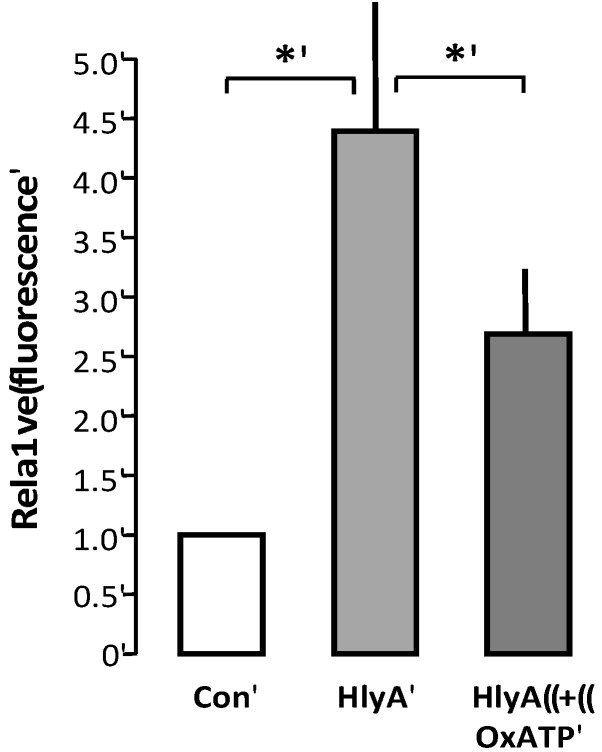
P2X receptor activation modulates HlyA-induced phagocytosis by THP-1 cells The figure shows the effect of oxATP 500 μM on the HlyA-induced erythrocyte phagocytosis by THP-1 cells. Bars show mean ± SEM, *n* = 6, asterisks indicate *p* < 0.05.

We therefore wanted to test whether P2 receptor blockage would also prevent phagocytosis of erythrocytes. This is, however, not very straightforward, as the THP-1 cells also express P2 receptors [12,13,14], which may potentially alter the ability to clear the damaged erythrocytes. The only way we can make that distinction is by pre-incubating the erythrocytes with the irreversible P2X blocker oxATP. OxATP was the least effective P2 receptor antagonist to reduce the erythrocyte PS exposure, but it nevertheless significantly reduced the HlyA-induced lysis of humane erythrocytes [2]. Since oxATP binds irreversible to the P2X receptors, we could add oxATP prior to the addition of HlyA to the erythrocytes, and wash the unbound away before adding the erythrocytes to the THP-1 cells. With this procedure, oxATP reduced the HlyA-induced phagocytosis of erythrocytes, compared to control (Figure 5).

## 3. Discussion

*E.* *coli* is the most prominent facultative Gram-negative bacterium in the colon and is the most common cause of urinary tract infections [15]. The exotoxin α-hemolysin (HlyA) is a known virulence factor in *E.* *coli* infections and there is an association between HlyA production and invasive uro-pathological strains [15]. HlyA-producing strains of *E.* *coli* have been shown to be more lethal than non-hemolytic strains in rodents after inoculation with *E.* *coli* intravenously, intraperitoneal, or in the urinary bladder [16,17,18,19,20,21,22]. This exotoxin causes severe cell damage by receptor-independent insertion into the plasma membrane as monomers and generating pores of ≈2 nm [23,24,25]. One single HlyA pore is considered sufficient to lyse one erythrocyte [26], which agrees very well with the finding that HlyA-induced hemolysis is amplified by extracellular ATP and following P2X receptor activation [2,4]. HlyA-producing *E.* *coli* strains are frequently isolated from patients with urinary tract infections, which, in severe cases, can ascend and cause pyelonephritis with serious risk of developing sepsis. Fulminate intravascular hemolysis is an infrequent but severe complication to sepsis [27]. Sepsis is associated with substantial anemia, which results from a combination of lysis and increased clearance of damaged erythrocytes by the immune system [27]. Despite the relative infrequency of visible intravascular hemolysis in sepsis, the recovery period from a septic episode is often complicated by anemia, which is partly caused by erythrocyte lysis [27]. Recent studies imply that free hemoglobin in plasma has a key position in determining the outcome of bacteremia. Mice that lacked hemoxygenase (Hmox^−/−^) showed marked increase in mortality after cecal ligation and puncture compared to controls [28]. This finding was substantiated through a number of experiments, including clear accumulation hemoglobin in the plasma of Hmox^−/−^ mice compared to both wild type and heterozygotes. This animal study is supported by a very recent clinical study, which signifies that a high free plasma-hemoglobin at hospital admission is associated with an increased 30-day mortality in septic patients [29]. This strongly suggests that it is of outmost importance to avoid free hemoglobin in plasma, as it is likely to result in a less favorable outcome of sepsis.

In this context, it is noteworthy that the hemolysis does not happen immediately after HlyA is inserted into the erythrocyte membrane. The process is a protracted process that consists of many steps. After the initial, receptor-independent insertion of HlyA into the membrane, there is an immediate increase in the [Ca^2+^]_i_, which is likely to be mainly mediated via Ca^2+^ permeation through the pore itself. Consequently, the [Ca^2+^]_i_ increases and activates the Ca^2+^-dependent K^+^ channels (K_Ca_3.1) and Cl^−^ channels (TMEM16A) [3]. Activation of the latter is responsible for the substantial volume reduction that precedes the eventual swelling and lysis. When HlyA is added at a concentration that cause 50% hemolysis over 60 min the erythrocytes shrinkage is maximal after ≈15 min [3]. The following swelling phase occurs when the influx of ions and water overcomes the conductance through the HlyA pore itself, and increasing stimulation of the ligand-gated ion-channels P2X as the environmental ATP increases. 

Here, we report that insertion of HlyA into the membrane of erythrocytes is a substantial trigger for recognition and phagocytosis of the damaged erythrocytes by THP-1 cells. Initially, we were able to confirm that THP-1 cells phagocytose erythrocytes, since the erythrocytes were localized intracellularly. Loading the erythrocytes with a fluorescent probe, phagocytosis could be detected as increases in THP-1 cell fluorescence. In this assay, it was clear that HlyA, similar to the positive control ionomycin, increased the detectable fluorescence in the THP-1 cells. It was striking that the fluorescence from the engulfed erythrocyte very quickly distributed throughout the cytosol of the THP-1 cells. This finding allowed us to confirm that the property of the fluorescence probe, by which the erythrocytes were primarily loaded, was preserved after the erythrocytes were engulfed by the THP-1 cells. This essentially means that the increase in fluorescence is not enhanced auto-fluorescence. Moreover, the fluorescence increase in the THP-1 cells is very unlikely to result from probe uptake from lysed erythrocytes. Firstly, the probe that is released from lysed erythrocytes will lack the -AM group required for significant cell loading. We have tested the addition of the same volume non-AM conjugated fluorescence probe, as is normally used for erythrocytes, and found that it did not cause a significant increase in the baseline fluorescence (data not shown). Secondly, we could not detect any fluorescence over the background of the supernatant from the preparation after 60 min of incubation with the calcein-loaded erythrocytes. Thirdly, the theoretic concentration of the fluorescence probe in the incubation solution had all erythrocytes lysed is of ≈7 × 10^−8^ M, assuming an intracellular fluo 4 concentration in the erythrocytes of 50 μM (assuming a 10-fold concentration of the probe), which is two orders of magnitude lower than what is used to load the cells with the AM form of the probe.

We found here that inhibition of the K_Ca_3.1 channels most effectively reduced the phagocytosis of erythrocytes damaged by HlyA. Inhibition of K_Ca_3.1 channels is known to inhibit both shrinkage and PS exposure in erythrocytes subjected to HlyA [3]. K_Ca_3.1 channel inhibition does not, however, prevent the HlyA cell damage. On the contrary, K_Ca_3.1 channel inhibition potentiates the HlyA-induced hemolysis by counteracting the shrinking process, which normally prolongs the kinetics of the hemolysis [3]. In line with this, it has been documented that erythrocytes from the K_Ca_3.1 knock-out mice are more prone to lyses after exposure to α-toxin from *Staphylococcus* *aureus*, [30]. These data indicate that either the reduction of cell size *per* *se* or the concomitant PS exposure is responsible for the increased phagocytosis of erythrocytes induced by HlyA. In terms of erythrocyte pathology, it is well recognized that any increment in [Ca^2+^]_i_ results in K_Ca_3.1-dependent cell shrinkage and PS exposure [30,31]. This process shows a close resemblance to the acute phase of apoptosis in nucleated cells and is, in some studies, referred to as *eryptosis* [32,33]. 

PS exposure is a known marker of both aged or damaged erythrocytes [6,31,32,33] and apoptotic nucleated cells [34]. Monocytes have receptors for phosphatidylserine and PS exposure has been substantiated as a signal for recognition and phagocytosis [35]. In line with this, our results clearly show that PS-covered beads are phagocytosed much more readily by the THP-1 cells compared to phosphatidylcholine-coated beads. Moreover, our data shows that inhibiting P2X receptors, which is known to reduce the cell damage inflicted by HlyA [2,4] also reduces the PS exposure. This is corroborated by previous reports on enhanced PS exposure on human erythrocytes upon P2X_7_ receptor activation [36]. PS exposure has been shown to be Ca^2+^-dependent and is likely to be associated with the sharp increase in [Ca^2+^]_i_ observed after addition of HlyA. Our very recent data show that this sharp increase in [Ca^2+^]_i_ is likely to be caused by the pore insertion itself, and that the P2X receptor activation is likely to account for the following slower [Ca^2+^]_i_ increment induced by HlyA [Ca^2+^]_i_ (data not shown). Determination of whether P2X receptor blockage on the erythrocytes also reduces the phagocytosis is not completely straightforward. In this context, it should be emphasized that monocytes express a variety of P2 receptors, at least P2Y_1_, P2Y_2_, P2Y_6_ P2Y_11–13,_ P2X_1_, P2X_4_, and P2X_7_ [37,38,39,40]. Our own results confirm this as we consistently find a sharp rise in [Ca^2+^]_i_ in the THP-1 cells upon addition of extracellular ATP, resembling a P2Y-type of [Ca^2+^]_i_ response. P2 receptor activation is essential in many monocyte/macrophage functions, such as the ATP concentration gradient responsible for the monocytic chemotaxis towards the infected cell, by stimulating the formation of pseudopodia [41]. In contrast, P2X_7_ is shown to inhibit phagocytosis at high concentrations and eventually, over time, causes apoptosis of the monocyte itself [42]. 

Therefore, we tested the effect of the only irreversible P2X antagonist we know to have a significant impact on HlyA-induced hemolysis: oxATP. Because of oxATP’s binding to the P2X receptors, we could wash away excess oxATP before the erythrocytes were added to the THP-1 cell preparation. We found that oxATP markedly reduced the HlyA-induced phagocytosis of human erythrocytes by the THP-1 cells. These data strongly suggest that P2X receptor inhibition not only prevent the cell damage associated with the pore insertion but also effectively prevent removal of the erythrocytes by phagocytotic cells. On this note, it is tempting to speculate that inhibition of the P2 receptor may have significant impact on the course of sepsis induced by HlyA-producing *E.* *coli*, not only the immediate cell damage but also the development of anemia during the recovery phase.

## 4. Experimental Section

### 4.1. Preparations of Erythrocytes

Blood samples were collected in EDTA-containing tubes, washed (1000*g*, 5 min, 4 °C) in 0.9% NaCl (*w*/*v*) followed by two washes in HEPES Buffered Salt solution (HBS) before the buffy coat was removed. The human blood was collected by venopuncture from healthy volunteers with evacuated blood collection tubes containing 63.1 mM K_2_EDTA. All human donors gave their written consent, and the study was approved by the Danish Scientific Ethics Committee (M20080027).

### 4.2. Toxin Purification

HlyA was purified from the supernatant from the *E.* *coli* strain ARD6 (serotype O6:K13:H1) grown in lysogeny broth (LB) medium in a process modified from the method described by Hyland *et* *al.*, [43]. A colony of *E.* *coli* ARD6 was transferred to 4 mL sterile LB medium and incubated overnight during constant swirl (37 °C, 200 rpm). The following morning, 1 mL of the overnight culture was transferred to 1 L sterile LB medium supplemented with 10 mM CaCl_2_ and incubated 4.5 h in a shaker (37 °C, 200 rpm). After incubation, cultures were centrifuged twice (2943*g*, 15 min, 4 °C) to pellet bacteria. The supernatant was sterile filtered (pore size 0.22 μm, Millipore, Bedford, MA, USA) and pH was adjusted to 4.5 (1 M malonic acid). HlyA was precipitated overnight with ethanol (25% *v*/*v*, 4 °C). The precipitate was centrifuged (17,300*g*, 30 min, 4 °C, Sorvall RC-5C, Thermo Scientific, Hvidovre, Denmark), and the pellet re-suspended in 6 M guanidine-HCl, precipitated for 60 min with ethanol (90% *v*/*v*, −20 °C) and centrifuged at 12,960*g*. The final precipitate containing HlyA was re-suspended in a TRIS-buffered 8 M guanidine-HCl solution with 10 mM DTT (pH 6.0). Although HlyA is the only detectable protein in this preparation [2], it is not free of LPS. For the experiments with PS exposure in the presence or absence of P2 antagonists, another preparation of HlyA was used, which was kindly provided by Professor Bhakdi (University of Mainz, Mainz, Germany).

### 4.3. Measurements of Hemolytic Activity

Washed human erythrocytes were suspended in HBS to produce an erythrocyte volume fraction of 1.25% in the final test solution. HlyA was added in increasing concentrations for 60 min at 37 °C under constant swirl (180 rpm) in 96-well plates. The experiment was terminated by centrifugation of the samples at 2325*g*, 3 min. The optical density of the supernatant was determined at 540 nm (OD_540_) in a plate reader (PowerWave Microplate Spectrophotometer, Biotek Instruments, Winooski, VT, USA), as a measure of the hemolytic activity. This procedure was used weekly to test the hemolytic activity of the HlyA preparation.

### 4.4. Cell Culture

Tamm-Horsefall protein 1 (THP-1) human, monocyte cell line from the American Type Culture Collection, Rockville, MD, USA was grown in cell flasks, on 25 mm diameter cover slips or in 96-well plates in Roswell Park Memorial Institute medium (RPMI-1640) supplemented with 10% fetal bovine serum (Gibco, Grand Island, NY, USA) and antibiotics (penicillin, 1 U mL^−1^ and streptomycin, 100 μg mL^−1^, Sigma-Aldrich, St. Louis, MO, USA). 

### 4.5. Phagocytosis Assay Microscope

Human erythrocytes were loaded with either calcein-AM (5 μM,) or fluo 4-AM (5 μM) for 60 min at 37 °C in HBS under constant swirl. The erythrocytes were washed twice to remove excess probe and exposed to HlyA, HBS (negative control) or ionomycin (1 μM, positive control) for 10 min in the dark. HlyA was added at a concentration, which resulted in 50% lysis after 60 min of incubation at 37 °C. After the short exposure to HlyA or ionomycin, the erythrocytes were washed twice and added to THP-1 cells grown on coverslips (50 μL of erythrocytes in suspension, 2.5% *v*/*v*) and incubated in the dark 60 min at 37 °C. Afterwards, the process was terminated by washing and placing the preparation on ice until inspection. The preparation was mounted in an inverted microscope (Nikon TE2000, Tokyo, Japan), and consecutive transmitted light and fluorescence images were obtained by either a Plan Apo 60X, NA1.2 Nikon for higher resolution images, or a Fluo 20X/0.75 NA Nikon for quantification of phagocytosis. For the analysis, the THP-1 cells were identified by the transmitted light images and fluorescence intensity (excitation 488 nm and emission >520 nm) was determined as the average intensity by placing regions of interest that covers ≈80% of the cell.

In the case of phagocytosis of coated beads, calcein-AM-loaded THP-1 cells (5 μM in 15 min) were incubated for 60 min with agarose beads, which were either phosphatidylserine or phosphatidylcholine-coated (Echelon Bioscience, Salt Lake City, UT, USA). Thereafter, the preparation was washed thoroughly to remove excess beads and placed for immediate inspection. After the preparation was mounted in the inverted microscope, consecutive pictures of fluorescence (excitation 488 nm, emission > 520 nm) and transmitted light (DIC) were taken using Plan Apo 60X, NA1.2 objective. The number of beads, taken up by the THP-1 cells, was counted in ImageJ, National Institutes of Health, Bethesda, MD, USA.

### 4.6. Phagocytosis Assay Plate Reader

As a supplement, phagocytosis was also determined via a fluorescence plate reader (Mithras LB 940, Berthold Technologies, Bad Wildbad, Germany). To this end, THP-1 cells were grown in collagen 1-coated 96-well plates (BD Biocoat Collagen 1, Rat tail, Thermo Fisher Scientific Inc., Waltham, MA, USA). Erythrocytes were loaded with calcein-AM (5 μM, 60 min at 37 °C in HBS in the dark), washed twice to remove extracellular probe and subjected to HlyA, HBS, or ionomycin for 10 min. After this procedure, the cells were washed twice, added the THP-1 cells in the 96-well plates (50 μL 2.5% erythrocytes to a total volume of 200 μL) and incubated for 60 min at 37 °C in HBS in the dark. The wells were thereafter washed twice in HBS and the fluorescence of each well was determined by excitation 488 nm and emission > 520 nm.

### 4.7. Live Cell [Ca^2+^]_i_ Imaging

For [Ca^2+^]_i_ measurements, erythrocytes were incubated with fluo 4-AM (5 μM, 60 min) and attached to glass coverslips by BD Cell-Tak™ and placed in an incubation chamber on a IMIC stage (TILL Photonics, Munich, Germany). The fluorophore was excited at 488 nm by a monochromator (PolychromeV, TILL Photonics), and the emission was collected >520 nm. The preparation was imaged with a 60×, 1.45 NA Plan Apo (Olympus, Gilleleje, Denmark) objective, and a charge coupled device camera (Sensicam qe, PCO, Kelheim, Germany). The entire set-up was delivered from Bio-Science ApS, Gilleleje, Denmark. The experiments were conducted at 37 °C.

### 4.8. Flow Cytometry

Flow cytometry analysis was performed on a FACSAria flow cytometer (BD Biosciences, Albertslund, Denmark), using BD FACSDiVa software for acquisition and analysis. Erythrocyte size was assessed using forward scatter (FSC) and density, using side scatter (SSC). All experiments were performed using a 100 μm nozzle and a standard sheet pressure of 20 psi. For all experiments, a suspension of erythrocytes containing approximately 10^6^ cells mL^−1^ was used. The flow rate was adjusted to keep cell count to 1000 events s^−1^. All experiments were conducted at 37 °C, 488 nm excitation/520 nm emission. For annexin V binding, 10,000 cells were investigated per experimental condition. The erythrocytes were incubated with HlyA (1 ng mL^−1^, resulting in 15%–20% shrinkage, but no cell lysis) in the presence or absence of PPADS, BBG, MRS2159 or oxATP for 10 min at room temperature. Hereafter, the cells were centrifuged for 3 min at 1000*g*, before cells were re-suspended in a solution containing annexin V and annexin V binding buffer (dilution, 1:50, Roche Diagnostics GmbH, Mannheim, Germany) and incubated in the dark for 10 min. The suspension was diluted 1/5 in a HBS, and the sample was run on the flow cytometer. 

### 4.9. Solutions and Materials

The HEPES-buffered salt solution (HBS) constituted of (in mM): [Na^+^] 138.0, [Cl^−^] 132.9, [K^+^] 5.3, [Ca^2+^] 1.8, [Mg^2+^] 0.8, [SO_4_^2−^] 0.8, [HEPES] 14, [glucose] 5.6, pH 7.4 at 37 °C. ATP-2',3'-dialdehyde (oxidized ATP), PPADS, TRAM-34, and MRS2159 were obtained from Sigma-Aldrich. Brilliant blue G (BBG) was purchased from INC Biomedicals Inc. (Aurora, OH, USA). Fluo 4-AM and calcein-AM was from Invitrogen (Taastrup, Denmark). Standard reagents were dissolved in distilled water and pH adjusted to 7.4 at 37 °C. BBG and TRAM-34 were both dissolved in DMSO to a final concentration of maximal 0.5% DMSO. Control experiments for possible DMSO effects alone were included.

### 4.10. Data Analysis and Statistics

Data are presented as mean ± SEM. The n value indicates number of experiments. In the hemolysis and phagocytosis experiments, the erythrocytes were isolated from a new donor for each experiment. The data were tested for normality by the Kolmogorov-Smirnov test. Significant differences were determined by paired or unpaired Student’s *t-test* or one-way Anova (Tukey *post* test) for multiple comparisons as appropriate. In both cases a *p*-value less than 0.05 was considered statistically significant.

## 5. Conclusions

In conclusion, the early cell damage inflicted by the pore-forming *E.* *coli* toxin HlyA efficiently marks erythrocytes for recognition and phagocytosis through monocytes. The initial erythrocyte shrinkage inflicted by HlyA is associated with PS exposure, which appears to be essential to the erythrocyte removal. PS exposure is hindered if either the HlyA-induced cell damage is prevented by P2X receptor inhibition or if cell shrinkage is prohibited. Our data are consistent with the notion that the erythrocyte shrinkage, which is the initial consequence of HlyA exposure, prevents acute lysis of the erythrocytes and is a strong signal for removal of the damaged erythrocytes from the blood stream, thereby reducing the risk of intravascular hemolysis.

## Supplementary Files

Supplementary File 1 Supplementary Information (PDF, 182 KB)
