# Supplementary material for: P2X Receptor-Dependent Erythrocyte Damage by α-Hemolysin from Escherichia coli Triggers Phagocytosis by THP-1 Cells
Source: Toxins (Basel). 2013 Mar 5;5(3):472–87. doi: 10.3390/toxins5030472 (PMC3705273; doi:10.3390/toxins5030472)
Supplement: Supplementary File 1 — Supplementary Information (PDF, 182 KB) [file toxins-05-00472-s001.pdf]

Supplementary Information

Table S1. Verification of erythrocyte phagocytosis by THP1-cells. (**A**) shows z-stacks of a preparation of THP-1 cells, which has been subjected to ionomycin-treated erythrocytes for 60 min. The green colour shows the fluorescence of a FITC-conjugated secondary antibody labelling anti-haemoglobin. The starting point for the z-stack is set to the centre of the erythrocyte in 0.2 µm steps in each direction. The pictures are obtained by the structural illumination function of the iMIC stage; (**B**) shows the corresponding DIC images of the preparation starting from the exact same start plane. The picture on the far is a merged side-view of florescence and DIC is shown; (**C**) shows representative picture of the increase in THP-1 cell fluorescence after exposure to ionomycin-treated fluo 4 loaded THP-1 cells; (**D**) shows the corresponding differential count of THP-1 cells grated either fluorescent positive or negative (mean ± SEM, *n* = 4–5) the asterisks indicate statistical significant reduction in fluorescence negative cells and increase in fluorescence positive cells by application of HlyA; (**E**) displays the increment in fluo-4 fluorescence by addition of extracellular ATP of when THP-1 cells loaded with fluo 4-AM. Each of the traces represent the average intensity over time for a region of interest placed over one cell. The bar graph shows the summation of the amplitude of the ATP-induced fluorescence increment; (**F**) shows the increment in fluorescence of THP1 cells when the cells are exposed to extracellular ATP. Here the THP1 cell-fluorescence stems from fluo 4-AM loaded erythrocytes, and illustrates that the fluorescence observed in the THP1 cells actually is the very same fluorescence probe that the erythrocytes were loaded with (*n* = 3). Each of the traces represents the average intensity over time for a region of interest placed over one cell.

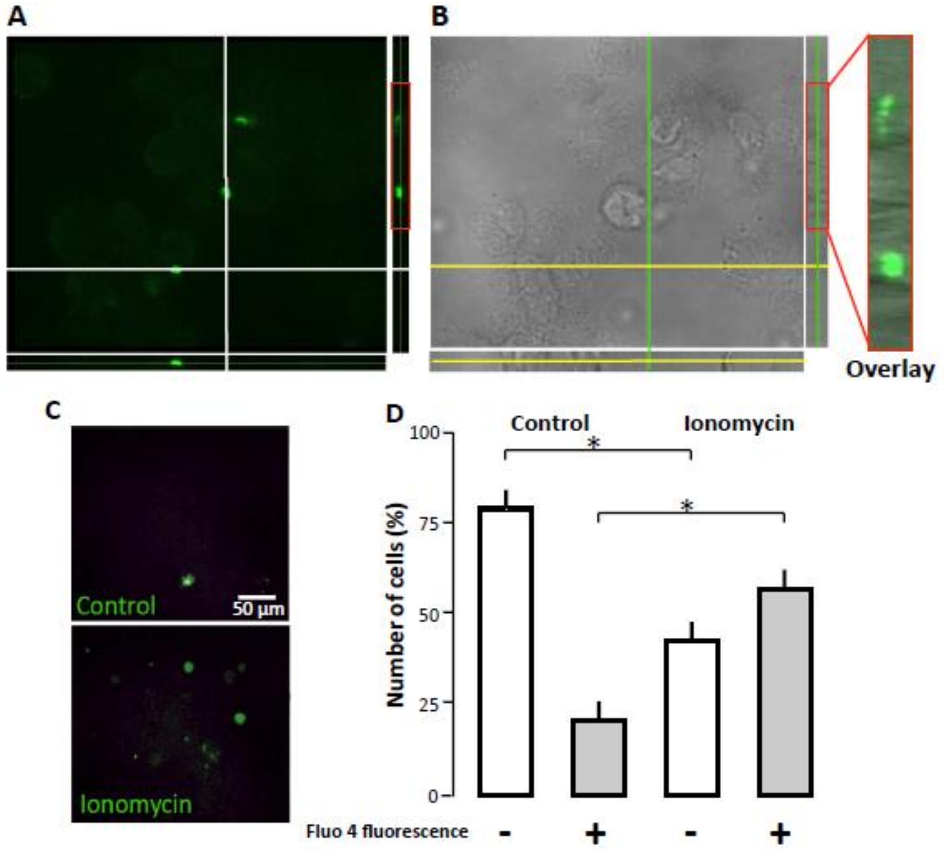

Figure S1. *Cont.*

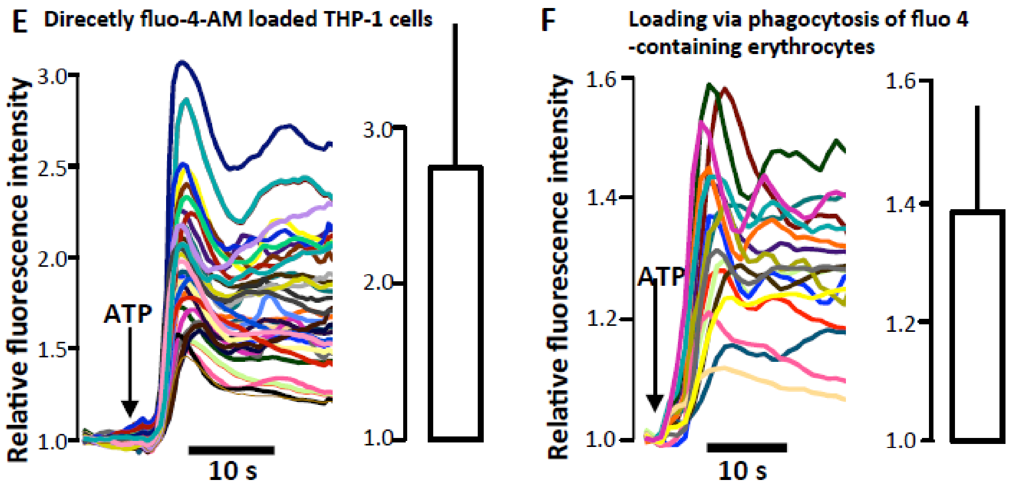

Figure S2. Phagocytosis of lipid-coated beads by THP-1 cells. (**A**) shows representative pictures of THP-1 cells exposed to either phosphatidylserine (PS) or phosphotidyl choline (PC) coated beads. Calcein loaded THP-1 cells were exposed to the beads for 60 min at 37 ℃; (**B**) summarize the data. Number of beads counted in Image J (PC = 47 cells, PS = 53, collected over 5 experimental days *p*-value below 0.05).

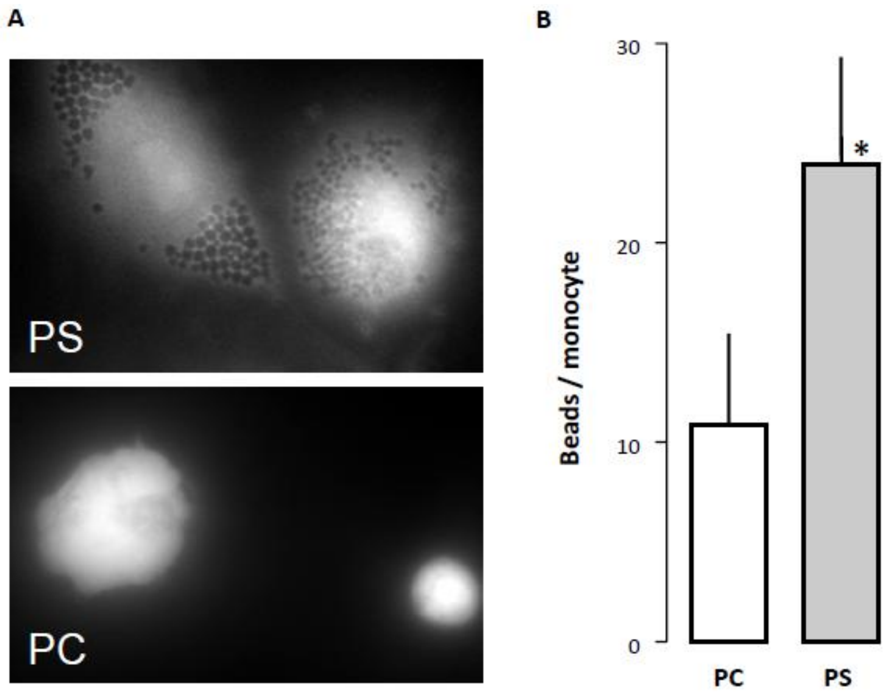

© 2013 by the authors; licensee MDPI, Basel, Switzerland. This article is an open access article distributed under the terms and conditions of the Creative Commons Attribution license (http://creativecommons.org/licenses/by/3.0/).
